# In Silico and In Vitro Evaluation of Calm Supplement Constituents on Antidepressant-Related Molecular Targets and Neuroplasticity-Associated Gene Expression

**DOI:** 10.3390/biomedicines14081749

**Published:** 2026-08-03

**Authors:** Lutfiye Karcioglu Batur, Ahsen Pektas, Buse Aslan, Nermin Akcali, Zeyneb Mustafa, Asli Ugurlu Bayarslan, Ebru Haciosmanoglu Aldogan, Savas Ustunova

**Affiliations:** 1 Department of Molecular Biology and Genetics, Biruni University, 34015 Istanbul, Türkiye; abayarslan@biruni.edu.tr; 2 Biruni University Research Center (B@MER), Biruni University, 34015 Istanbul, Türkiye; 3 Kiperin Pharmaceutical and Food Industry and Trade Ltd., 34307 Istanbul, Türkiye; ahsennzerin@gmail.com (A.P.); busseaaslan@gmail.com (B.A.); zeynebmust54@gmail.com (Z.M.); 4 Department of Medical Genetics, Faculty of Medicine, Bandirma Onyedi Eylul University, 10250 Balıkesir, Türkiye; nakcali@biruni.edu.tr; 5 Department of Biophysics, Faculty of Medicine, Istanbul University-Cerrahpasa, 34320 Istanbul, Türkiye; ebru.aldogan@iuc.edu.tr; 6 Department of Physiology, Faculty of Medicine, Bezmialem Vakif University, 34093 Istanbul, Türkiye; sustunova@bezmialem.edu.tr

**Keywords:** calm supplement, SH-SY5Y cells, molecular docking, *NTRK2*/TrkB, neuroplasticity

## Abstract

**Background**: Depression-related mechanisms involve not only monoaminergic neurotransmission but also neurotrophic signaling, synaptic plasticity and intracellular survival pathways. **Materials and Methods**: In this study, the potential neuropharmacological properties of Calm, a multi-component supplement product, were evaluated using in silico and in vitro approaches. Molecular docking analyses were performed to estimate the binding profiles of Calm-derived constituents and reference antidepressants, sertraline and fluoxetine, against SLC6A4, MAOA, AKT1 and NTRK2. In parallel, SH-SY5Y human neuroblastoma cells were treated with Calm, and MTT-based metabolic activity and the relative expression levels of *BDNF, NTRK2* and *SYN1* were assessed after 24 h. **Results**: Docking results showed that several Calm constituents, particularly apigenin, rosmarinic acid, xanthohumol and valerenic acid, exhibited favorable predicted interactions with SLC6A4 and NTRK2. Calm treatment increased MTT signal at low concentrations, with significant increases observed at 10, 30, and 60 µg/mL. However, 24 h exposure to 10 µg/mL Calm significantly reduced *BDNF*, *NTRK2* and *SYN1* expression levels. **Conclusions**: These findings suggest that Calm constituents may interact with antidepressant-related molecular targets and modulate neuroplasticity-associated transcriptional markers. However, the results should be considered preliminary and require protein-level and functional validation.

## 1. Introduction

Depression and related neuropsychiatric disorders are associated not only with alterations in monoaminergic neurotransmission, but also with impairments in neuronal plasticity, neurotrophic support, synaptic remodeling and intracellular survival signaling [1,2,3,4,5]. In the conventional pharmacological approach, selective serotonin reuptake inhibitors exert their effects mainly by increasing synaptic serotonin levels through the serotonin transporter SLC6A4 (SERT) [6,7,8]. Antidepressants such as sertraline and fluoxetine are well-known examples of this mechanism [9]. However, it is also accepted that the antidepressant response cannot be explained solely by an acute increase in serotonin. Rather, longer-term effects are associated with the Brain-derived neurotrophic factor/tropomyosin receptor kinase B (BDNF/TrkB) protein signaling axis [10,11,12], AKT-mediated intracellular signaling pathways [13] and molecular adaptations related to synaptic plasticity [14].

In this context, monoamine oxidase A (MAOA), which plays a role in monoamine metabolism; SLC6A4, one of the key regulators of serotonergic transmission; AKT1, a central element in cell survival and plasticity signaling; and Neurotrophic Receptor Tyrosine Kinase 2 (NTRK2/TrkB), the high-affinity receptor of BDNF, are considered biologically relevant targets in antidepressant-related cellular responses [15,16]. In particular, NTRK2 is noteworthy because it forms a functional bridge between monoaminergic mechanisms and neurotrophic/synaptic plasticity responses [11,12,17]. Examining these targets may therefore help clarify whether neuroactive compounds influence molecular pathways related to antidepressant response and neuroplasticity.

Herbal or multi-component supplement products may contain various active molecules that can simultaneously affect different biological targets. Components present in Calm, such as valerenic acid (Valerian extract), harmine (Passionflower extract), apigenin (Chamomile extract), 5-hydroxytryptophan, rosmarinic acid (Lemon Balm extract), xanthohumol (Hop extract), safranal (Saffron extract) and pyridoxal-5-phosphate, are considered among molecules associated in the literature with neuromodulation [18,19,20,21,22], antioxidant response [23,24,25,26,27], monoaminergic regulation [28,29,30,31,32] and neuronal functions [33,34,35,36,37,38]. However, it remains unclear how such multi-component products may interact with antidepressant-related targets at the cellular level and whether they affect gene expression responses related to neuroplasticity [39].

In this study, we evaluated the potential neuropharmacological effects of Calm (Kiperin, Türkiye) using in vitro and in silico approaches. Positioned primarily as a Passiflora-based multi-component supplement, Calm is formulated to combine the synergistic effects of various herbal extracts and nutritional cofactors. The active ingredients of the supplement include Passiflora, Valerian, German Chamomile, Lemon Balm, Hops, and Saffron extracts. Additionally, the formulation is supported with 5-Hydroxytryptophan (5-HTP), L-Tryptophan, and Vitamin B6 to enhance its targeted biological activities. First, we examined the possible interactions of Calm constituents and the reference antidepressants Sertaline/sertraline and Fluoxetine/fluoxetine with SLC6A4, MAOA, AKT1 and NTRK2 proteins by molecular docking analysis. These targets were selected because they are associated with serotonergic transport [6], monoamine metabolism [17], intracellular plasticity/survival signaling [13] and BDNF/TrkB-mediated neurotrophic signaling [10], respectively. In the second stage, based on the NTRK2 axis that emerged as biologically relevant in the docking analyses, we evaluated the expression levels of *BDNF*, *NTRK2*, and (Synapsin I) *SYN1* genes in SH-SY5Y human neuroblastoma cells. Although SH-SY5Y cells are derived from human neuroblastoma, they are widely used as a neuronal-like in vitro screening model for investigating neuroprotection, oxidative stress-related neuronal injury, neurite outgrowth, and neuroactive compound responses [40,41]. On the other hand, the gene panel was selected to monitor BDNF/TrkB-mediated neurotrophic signaling and possible cellular responses associated with synaptic plasticity. Thus, rather than demonstrating the direct therapeutic efficacy of Calm, this study provides a preliminary in silico and in vitro assessment of its potential interactions with antidepressant-related molecular targets and its effects on neuroplasticity-associated gene expression markers.

## 2. Materials and Methods

### 2.1. Preparation and Composition of the Dietary Supplement Calm

The commercial multi-component dietary supplement, Calm, was commercially obtained from Kiperin Pharmaceutical and Food Industry and Trade Ltd., Istanbul, Türkiye. According to the manufacturer’s specifications, each capsule of the supplement contains a standardized mixture of the following active ingredients: Valerian extract (140 mg), Passiflora extract (140 mg), German Chamomile (*Matricaria chamomilla*) extract (90 mg), 5-Hydroxytryptophan (5-HTP) (90 mg), Lemon Balm (*Melissa officinalis*) extract (90 mg), L-Tryptophan (90 mg), Hops (*Humulus lupulus*) extract (30 mg), Saffron (*Crocus sativus*) extract (10 mg), and Vitamin B6 (1.4 mg). For experimental use, the capsule contents were obtained and prepared by dissolving in distilled water immedi-ately prior to vehicle administration.

### 2.2. Cell Culture

SH-SY5Y human neuroblastoma cells (SH-SY5Y, ATCC; cat.no CRL-2266; Manassas, VA, USA) were cultured in Dulbecco’s Modified Eagle Medium (DMEM) (Gibco, Thermo Fisher Scientific, Waltham, MA, USA) supplemented with 10% fetal bovine serum (Gibco, Thermo Fisher Scientific, Waltham, MA, USA) and 1% antibiotics (penicillin-streptomycin) (Gibco, Thermo Fisher Scientific, Waltham, MA, USA) and maintained at 37 °C in a humidified incubator with 5% CO_2_ (Panasonic Healthcare Co., Ltd., Tokyo, Japan).

Cells were seeded into sterile 96-well plates at a density of 1 × 10^4^ cells/well and incubated for 24 h. After incubation, the medium was removed and replaced with fresh medium containing Calm at concentrations ranging from 10 to 200 µg/mL, followed by a further 24 h incubation (n = 4). Cell viability was assessed using the 3-(4,5-dimethylthiazol-2-yl)-2,5-diphenyltetrazolium bromide (MTT) assay (EcoTech Biotechnology, Istanbul, Türkiye). After 24 h of treatment, 10 µL of MTT solution (5 mg/mL prepared in PBS) was added to each well and incubated for 4 h at 37 °C under 5% CO_2_. Subsequently, the medium was carefully removed, and the resulting formazan crystals were dissolved in 100 µL of dimethyl sulfoxide (DMSO). Absorbance was measured at 570 nm using a microplate reader (BioTek Instruments, Winooski, VT, USA). The obtained absorbance values were converted into relative percentage (%) values by setting the control group as 100%.

### 2.3. Molecular Docking Analysis

Molecular docking analysis was performed using AutoDock Vina version 1.2.0 [42,43] to evaluate the potential binding interactions between selected Calm-derived bioactive compounds and the NTRK2, AKT1, MAOA and SLC6A4 proteins. The three-dimensional structures of the proteins were obtained from the Protein Data Bank using the PDB IDs 4AT3, 1H10, 2Z5Y and 5I6X, respectively. Protein preparation was carried out using AutoDockTools version 1.5.7. During this step, water molecules and the co-crystallized ligand were removed, and polar hydrogen atoms were added. Also, Kollman charges were assigned, and the prepared protein structures were converted into PDBQT format.

The binding site was defined based on the coordinates of the native ligand present in the crystal structure. The grid box was centered for each protein. Sertraline and fluoxetine were used as reference antidepressant compounds, while eight (8) compounds, namely valerenic acid, harmine, apigenin, safranal, 5-hydroxytryptophan, rosmarinic acid, xanthohumol, and pyridoxal-5-phosphate were included as Calm-derived ligands, representing the primary active constituents of the supplement’s botanical and nutritional ingredients. Ligand structures were converted from SDF format to PDBQT format using Open Babel version 3.1.1 [44].

Docking simulations were performed using the default AutoDock Vina version 1.2.0 parameters, with the exhaustiveness value set to 8. For each ligand, the pose with the lowest binding energy was selected as the best docking pose. Binding affinities were recorded as binding energy values in kcal/mol, with more negative values indicating stronger predicted ligand–protein interactions. Docking results and ligand–protein interactions were visualized using PyMOL version 3.1.

### 2.4. Total RNA Extraction and cDNA Library

Total RNA was isolated using TRIzol reagent (Invitrogen, Thermo Fisher Scientific, Waltham, MA, USA). Cells were seeded into sterile 6-well plates at a density of 4 × 10^5^ cells/well and incubated for 24 h. Following incubation, cells were treated with 10 µg/mL of Calm, Sertaline (Zoloft^®^, Pfizer Inc., New York, NY, USA) or Fluoxetine (Prozac^®^, Eli Lilly and Company, Indianapolis, IN, USA) for 24 h. After treatment, plates were placed on ice and each well was washed twice with ice-cold PBS (pH 7.4). PBS was removed, and 1 mL of TRIzol reagent was added to each well. Cells were then transferred to nuclease-free microcentrifuge tubes.

Subsequently, 250 µL of chloroform was added, samples were vigorously shaken for 15 s and incubated at room temperature for 3 min. Samples were centrifuged at 12,000× *g* for 15 min at 4 °C. The aqueous phase was transferred to new tubes and mixed with 500 µL of isopropanol, followed by incubation at room temperature for 10 min. After centrifugation at 12,000× *g* for 10 min at 4 °C, the RNA pellet was washed with 75% ethanol, air-dried under a laminar flow hood, and resuspended in 80 µL of nuclease-free distilled water.

RNA concentration and purity were evaluated using an ND-2000c NanoDrop spectrophotometer (Thermo Fisher Scientific, Waltham, MA, USA), and RNA concentrations were normalized. cDNA synthesis was performed using the OneScript Plus cDNA Synthesis Kit (Applied Biological Materials Inc. (ABM), Richmond, BC, Canada) according to the manufacturer’s instructions.

### 2.5. Gene Expression

RT-qPCR experiments were performed using BlasTaq 2X qPCR MasterMix (Applied Biological Materials Inc. (ABM), Richmond, BC, Canada) and *BDNF*, *NTRK2* and *SYN1* primer pairs (Table 1). Glyceraldehyde-3-phosphate dehydrogenase (GAPDH) was used as the reference gene. Each reaction contained 1 µg cDNA and 250 nM of forward and reverse primers.

Thermal cycling was initiated with an initial denaturation step at 95 °C for 3 min, followed by 40 cycles of 95 °C for 15 s and 60 °C for 1 min. Amplification was carried out using a CFX96 Real-Time System C1000 Touch Thermal Cycler (Bio-Rad Laboratories, Hercules, CA, USA). Relative gene expression levels were calculated using the 2^−ΔΔCt^ method. Each experiment was performed six times (n = 6), and a no-template control (NTC) was included in each run.

### 2.6. Statistical Analysis

All statistical analyses were performed using GraphPad Prism version 11.0 (GraphPad Software, Boston, MA, USA). Prior to parametric testing, the normality of the data distribution was assessed and confirmed using the Shapiro–Wilk test. Data are presented as mean ± standard deviation (SD). Differences among groups were analyzed using one-way analysis of variance (ANOVA) followed by Tukey’s multiple comparisons test. All statistical comparisons were performed relative to the control group. The *p* value of < 0.05 was considered statistically significant.

## 3. Results

### 3.1. MTT Assay

**Figure 1:**
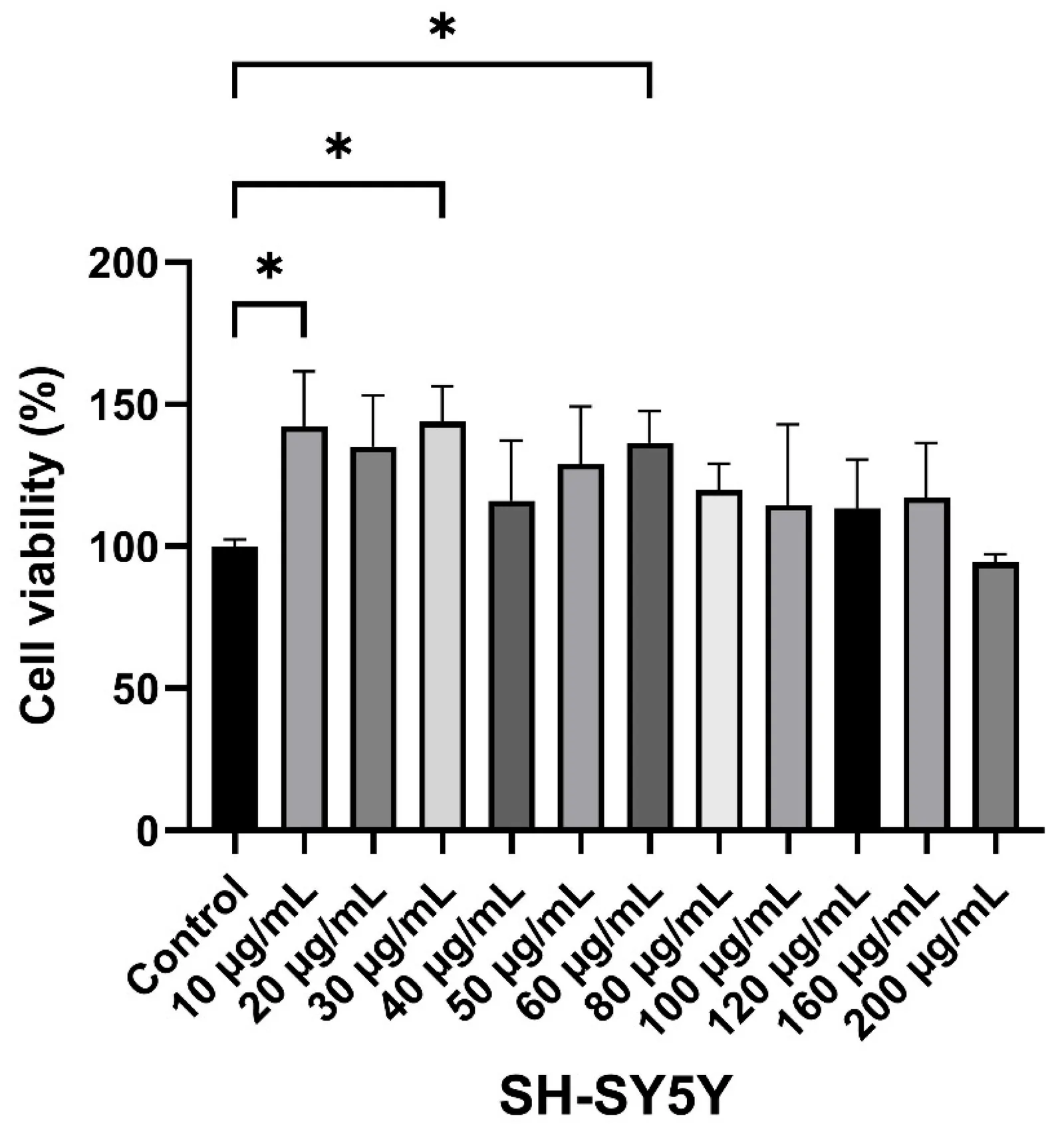
Effects of Calm on MTT assay in SH-SY5Y cells following 24 h treatment with increasing concentrations of Calm (10–200 µg/mL). Results are expressed as percentage relative to the control group set as 100%. Data are presented as mean ± SD (n = 4). Statistical analysis was performed relative to the control group (* *p* < 0.05).

MTT results following Calm treatment are presented relative to the control group (set as 100%). A significant increase in MTT signaling was observed at 10 µg/mL (142.3 ± 15.8%, *p* < 0.05), 30 µg/mL (144.0 ± 10.0%, *p* < 0.05) and 60 µg/mL (136.5 ± 9.6%, *p* < 0.05) concentrations compared to the control group (Figure 1). Although increases in viability were also observed at other concentrations, these were not statistically significant.

At higher concentrations, particularly 200 µg/mL (94.2 ± 2.5%), MTT signaling slightly decreased compared to control; however, this reduction was not statistically significant.

### 3.2. Molecular Docking

Molecular docking analysis was performed to evaluate the predicted binding profiles of the reference antidepressants, sertraline and fluoxetine, along with Calm-derived bioactive compounds, against selected neuropharmacological targets: SLC6A4, MAOA, AKT1, and NTRK2. The binding energies and predicted ligand–protein interactions are summarized in Table 2.

**Figure 2:**
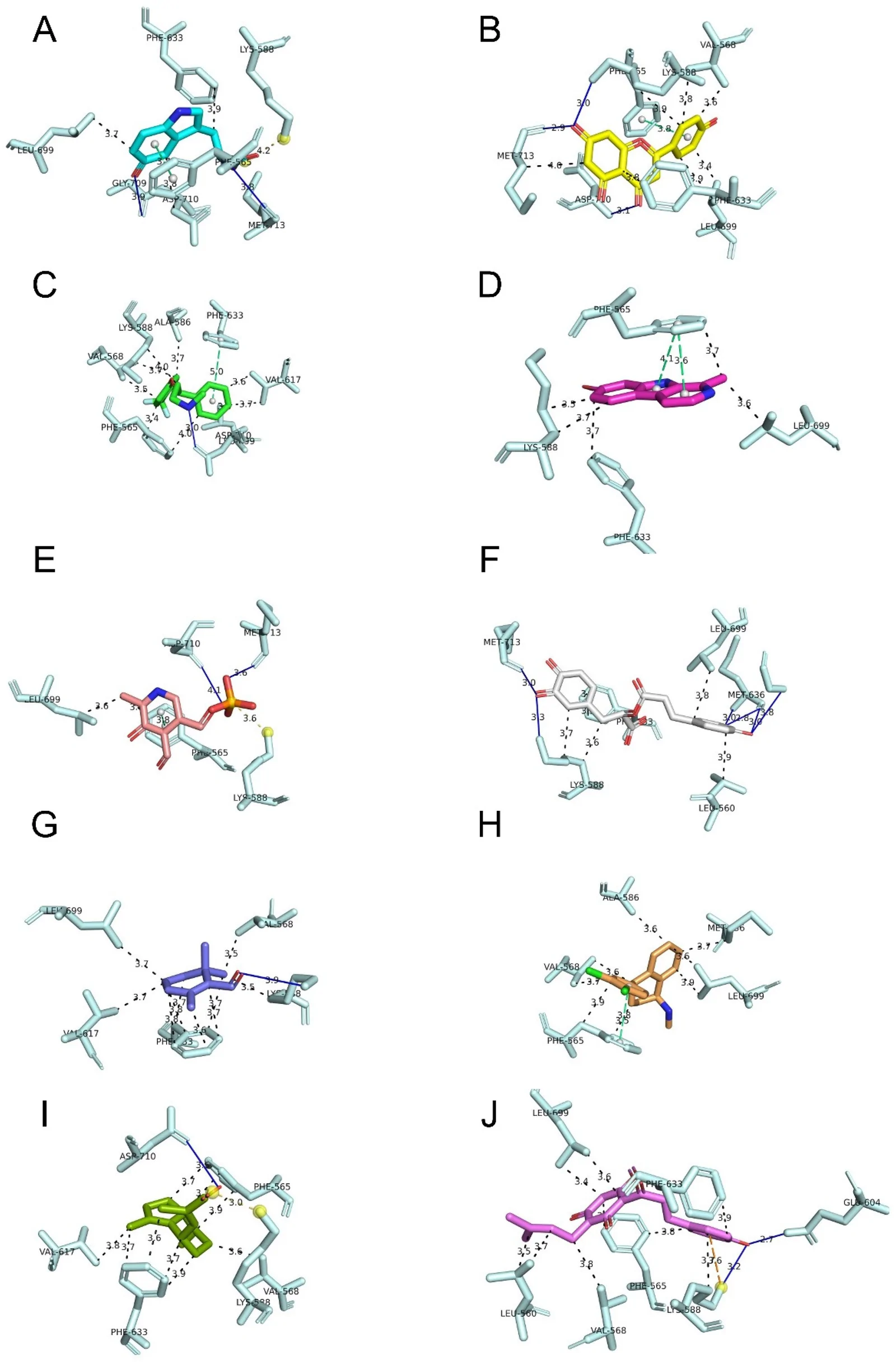
Predicted binding poses and ligand–residue interactions of reference antidepressants and Calm-derived compounds with NTRK2/TrkB. Representative docking interaction poses of Calm-derived compounds and reference antidepressants within the NTRK2/TrkB binding region are shown. (**A**) 5-hydroxytryptophan, (**B**) apigenin, (**C**) fluoxetine, (**D**) harmine, (**E**) pyridoxal-5-phosphate, (**F**) rosmarinic acid, (**G**) safranal, (**H**) sertraline, (**I**) valerenic acid and (**J**) xanthohumol. Only amino acid residues predicted to interact with each ligand are displayed. Interaction types are indicated by colored lines: blue, hydrogen bonds; black, hydrophobic interactions; yellow, salt bridges; orange, π-cation interactions; and green, π-stacking interactions. Docking results and ligand–protein interactions were visualized using PyMOL version 3.1.

In general, the compounds showed target-dependent binding profiles. Among the evaluated proteins, SLC6A4 and NTRK2 displayed more favorable binding energies compared with MAOA and AKT1. This tendency was especially evident for several Calm-derived compounds, suggesting that serotonergic transport and BDNF/TrkB-related signaling may be more relevant molecular contexts for these predicted interactions (Figure 2).

Among the reference antidepressants, sertraline showed strong predicted binding to both SLC6A4 and NTRK2, with binding energies of −9.7 kcal/mol and −10.2 kcal/mol, respectively. Fluoxetine also showed favorable interactions with these targets, particularly with SLC6A4 (−9.2 kcal/mol) and NTRK2 (−8.7 kcal/mol). These findings are in line with the pharmacological relevance of serotonergic mechanisms for these reference drugs.

Among the Calm-derived compounds, apigenin showed one of the most favorable overall binding profiles, with strong predicted interactions with SLC6A4 (−10.0 kcal/mol) and NTRK2 (−9.6 kcal/mol). Rosmarinic acid and xanthohumol also showed favorable binding to these two targets. Rosmarinic acid displayed binding energies of −9.0 kcal/mol for SLC6A4 and −9.4 kcal/mol for NTRK2, while xanthohumol showed binding energies of −9.2 kcal/mol and −8.7 kcal/mol, respectively. Valerenic acid also showed a strong predicted interaction with NTRK2 (−9.3 kcal/mol), together with a favorable interaction with SLC6A4 (−8.2 kcal/mol).

For MAOA, the strongest predicted interaction among the Calm constituents was observed with harmine (−8.6 kcal/mol), followed by apigenin (−8.3 kcal/mol) and 5-hydroxytryptophan (−7.8 kcal/mol). This result is noteworthy since MAOA is directly involved in monoamine metabolism. However, the overall binding profile of Calm constituents toward MAOA was less consistent than that observed for SLC6A4 and NTRK2.

In contrast, AKT1 showed comparatively weaker predicted binding energies across most ligands. The strongest Calm-derived interactions with AKT1 were observed for xanthohumol (−5.8 kcal/mol), apigenin (−5.7 kcal/mol) and rosmarinic acid (−5.6 kcal/mol). These values were lower than those observed for SLC6A4, MAOA and NTRK2.

### 3.3. Relative Expressions of BDNF, NTRK2 and SYN1 Genes

Since *NTRK2* showed favorable predicted binding interactions with several Calm-derived compounds and is directly linked to the BDNF/TrkB signaling axis, subsequent gene expression analysis focused on *NTRK2* and related neuroplasticity-associated markers. Accordingly, *BDNF*, *NTRK2*, and *SYN1* were evaluated to examine possible changes in neurotrophic and synaptic plasticity-related responses.

**Figure 3:**
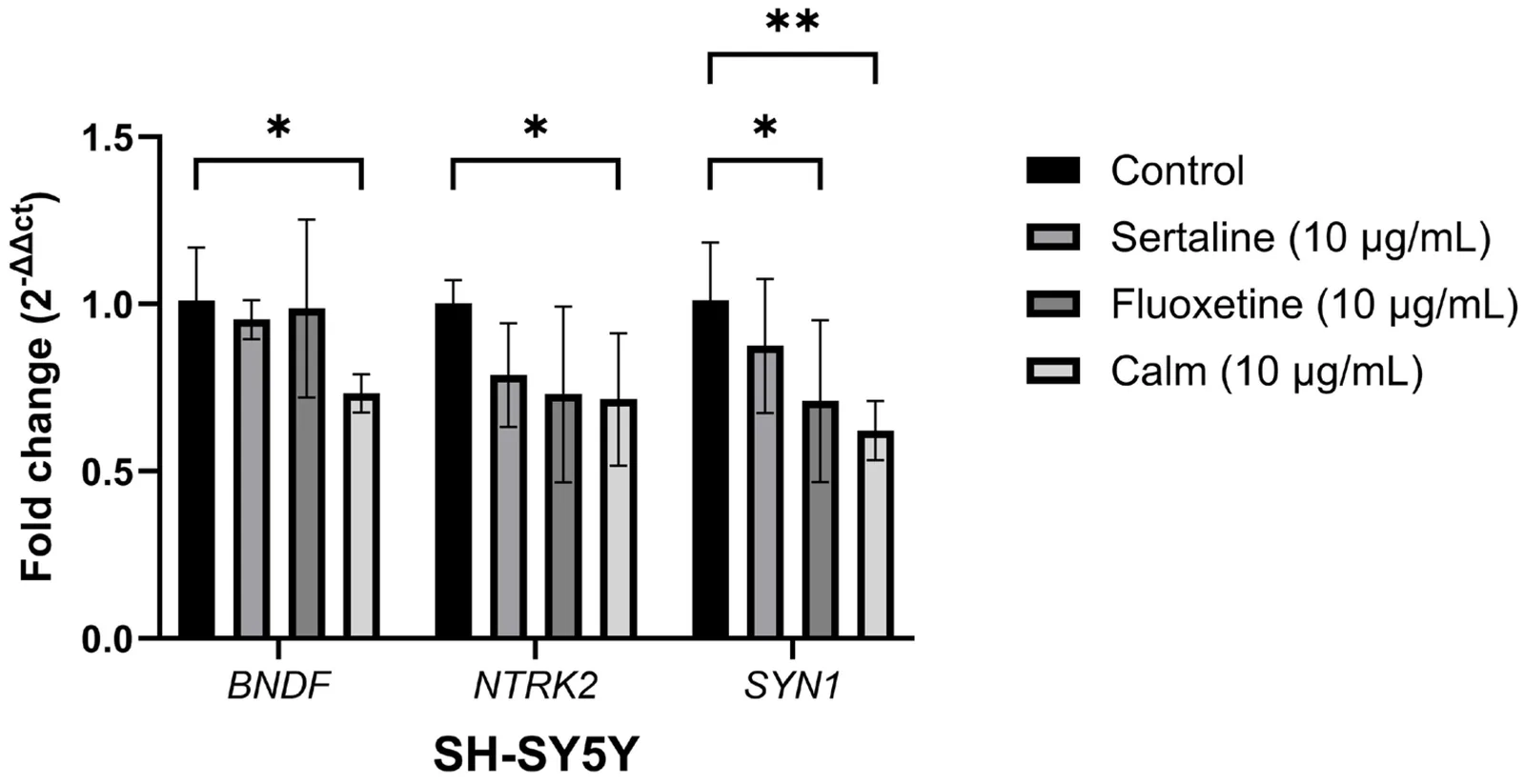
Gene expressions of *BDNF*, *NTRK2* and *SYN1* genes. Relative mRNA expression levels of *BDNF*, *NTRK2* and *SYN1* were determined in SH-SY5Y cells following treatment with Sertaline, Fluoxetine, and Calm (10 µg/mL) for 24 h. Expression levels were calculated using the 2^−ΔΔCt^ method. Data are expressed as mean ± SD from independent experiments (n = 6). Statistical significance was evaluated compared to the control group (* *p* < 0.05, ** *p* < 0.01).

Relative expression levels of *BDNF*, *NTRK2* and *SYN1* genes were evaluated following treatment with Sertaline (10 µg/mL), Fluoxetine (10 µg/mL) and Calm (10 µg/mL) (Figure 3). No significant changes were observed in *BDNF* expression in the Sertaline and Fluoxetine groups compared to the control. However, Calm treatment significantly reduced *BDNF* expression (~0.7-fold, *p* < 0.05) after 24 h. Similarly, *NTRK2* expression showed a decreasing trend in all treatment groups, with a statistically significant reduction observed only in the Calm-treated group (~0.7-fold, *p* < 0.05). For *SYN1*, a significant decrease was detected in both the Calm (~0.6-fold, *p* < 0.01) and Fluoxetine groups (~0.7-fold, *p* < 0.05), while the reduction in the Sertaline group was not statistically significant.

## 4. Discussion

In this study, the potential neuropharmacological effects of Calm, a multi-component supplement product, were evaluated through molecular docking analysis and SH-SY5Y cell line-based in vitro experiments. The findings revealed that some Calm constituents showed favorable predicted binding profiles with antidepressant-related targets, particularly SLC6A4 and NTRK2. In parallel, Calm treatment increased MTT-based cell viability/metabolic activity at low concentrations. However, 24 h exposure to 10 µg/mL Calm caused a significant decrease in *BDNF*, *NTRK2*, and *SYN1* gene expression levels. These results indicate that Calm constituents may interact with molecular targets associated with serotonergic transmission and neurotrophic signaling.

The molecular docking results appear to be compatible with the current view suggesting that antidepressant-related responses are linked not only to monoaminergic mechanisms but also to pathways associated with neurotrophic and synaptic plasticity [11,17]. In line with this broader perspective, non-monoaminergic agents such as esketamine, which act primarily through NMDA receptor modulation, have also demonstrated efficacy in depression-related conditions, further supporting the notion that antidepressant-related mechanisms extend beyond classical monoaminergic pathways [45].

Although sertraline and fluoxetine are mainly associated with SLC6A4/SERT-mediated serotonin reuptake inhibition, both reference drugs showed favorable predicted binding affinities with SLC6A4 in the present study. In addition, sertraline and fluoxetine also showed notable predicted interactions with NTRK2. Current studies indicate that TrkB may play an important mediating role in antidepressant drug action and may link monoaminergic modulation with BDNF-dependent neuroplasticity. In this respect, the favorable docking profiles observed for Calm constituents such as apigenin, rosmarinic acid, xanthohumol, and valerenic acid also support the possibility that these compounds may interact with targets associated with serotonergic and neurotrophic signaling.

Among the Calm constituents, apigenin showed one of the strongest predicted binding profiles, particularly for SLC6A4 and NTRK2. This result is consistent with previous studies describing apigenin as a neuroactive flavonoid with antioxidant, anti-inflammatory, and neuroprotective properties [25,36]. Rosmarinic acid also showed favorable predicted interactions with both SLC6A4 and NTRK2. This result is also noteworthy because rosmarinic acid has previously been associated with antioxidant activity, neuroprotective effects, and antidepressant/anxiolytic-like mechanisms [19,34]. Similarly, xanthohumol [33] and valerenic acid [20,38] also showed relatively favorable binding profiles, and there are reports indicating that these two compounds may affect neuronal or neuromodulatory processes.

The docking profile toward MAOA showed a more compound-specific pattern. Among the Calm constituents, the strongest predicted interaction with MAOA was observed for harmine. Considering the relationship of harmine with MAOA inhibition and monoaminergic regulation, this observation is consistent with the exploratory nature of the docking analysis. Apigenin and 5-hydroxytryptophan also showed relatively favorable predicted binding with MAOA. These findings are important because monoamine metabolism is one of the molecular contexts associated with antidepressant-related responses [1,2,30]. In contrast, AKT1 showed weaker binding energies for most ligands compared with SLC6A4, MAOA, and NTRK2. Although AKT signaling is an important pathway in depression-related neuroplasticity and cell survival [13,16], the present docking results suggest that AKT1 is less likely to be a primary direct binding target for Calm constituents. Although AKT1 showed weaker predicted binding profiles, the interconnected nature of intracellular signaling pathways means that indirect modulation of AKT-related signaling cannot be excluded without protein-level validation. However, mechanistic studies conducted at the protein level are needed to support these inferences.

MTT analysis showed that Calm increased cell viability/metabolic activity at concentrations of 10, 30, and 60 µg/mL, whereas it did not produce a significant increase at higher concentrations and showed a slight but non-significant decreasing tendency at 200 µg/mL, a concentration well above the physiological exposure range. The increase in cell viability observed at concentrations below 200 μg/mL is considered to reflect a mild hormetic response, whereas the loss of this stimulatory effect at concentrations of 200 μg/mL and above may be attributable to saturation of the protective response rather than overt cytotoxicity. As a limitation of this study, higher concentrations above 200 μg/mL should be tested in future studies to determine the true cytotoxic threshold. This pattern indicates that Calm produces a concentration-dependent cellular response in SH-SY5Y cells. However, it should also be considered that the increase in MTT signal reflects mitochondrial/metabolic activity [46]. To evaluate this result from a neuroprotective perspective, additional damage models such as oxidative stress-mediated injury, apoptosis, and mitochondrial dysfunction should also be examined.

Consistent with its predicted molecular interactions, Calm treatment induced transcriptional changes in *BDNF*, *NTRK2*, and *SYN1* after 24 h, which may represent an early adaptive response associated with the modulation of neurotrophic and synaptic plasticity pathways [10,17]. However, this finding may reflect a short-term transcriptional adaptation rather than a direct suppression of neurotrophic function [47]. Antidepressant-related neuroplastic responses often depend on treatment duration, cellular context, and post-transcriptional or protein-level regulations [10,11,14,16,17]. Therefore, the 24 h treatment period may have captured an early regulatory phase rather than a stable and long-term neuroplastic response. In addition, the NTRK2–ligand interaction predicted by docking does not mean that *NTRK2* mRNA expression should increase. Receptor activation, receptor stabilization, phosphorylation status, and downstream signal transduction may occur independently of an increase at the transcript level [10,11,17].

The decrease in SYN1 expression suggests that short-term Calm exposure may affect synaptic plasticity-related markers differently from classical long-term antidepressant adaptation. Although SYN1 is not a direct component of the BDNF/TrkB receptor complex, it is rather a marker associated with synaptic vesicle regulation, synaptic function, and plasticity [48]. Therefore, the decrease in *SYN1* expression may be considered a downstream transcriptional response related to acute treatment conditions. Interestingly, Fluoxetine also significantly reduced *SYN1* expression, whereas Sertaline also reduced gene expression but did not cause a statistically significant change. This suggests that even reference antidepressants may produce gene-specific and compound-specific responses in SH-SY5Y cells under short-term in vitro conditions.

SH-SY5Y cells are a practical neural cell line widely used for investigating processes such as neuroactive compounds, neuroprotection, oxidative stress-related neuronal injury, and neurite outgrowth [40,41]. However, SH-SY5Y cells are derived from human neuroblastoma and do not fully reflect the complexity of mature neurons, glial cell interactions, CNS location-based signaling events, or CNS-derived diseases. Therefore, the present findings should be regarded as preliminary yet promising data demonstrating that Calm constituents are capable of interacting with molecular targets associated with serotonergic transmission and neuroplasticity-related pathways, and should be acknowledged as providing a strong scientific basis for further investigation. On the other hand, considering that BDNF/TrkB-related responses are strongly regulated through protein levels and phosphorylation status, future studies should also evaluate BDNF protein levels, total and phosphorylated TrkB, as well as downstream signaling markers such as p-AKT and p-ERK. Finally, Calm is a multi-component product, and the present experimental design does not allow the contribution of each individual compound to the total response to be distinguished. Therefore, the increase in cell viability/metabolic activity should not be directly interpreted as neuroprotection.

Docking scores in this study reflect theoretical, static binding predictions and were used solely to guide target selection for the subsequent functional experiments; they do not constitute direct evidence of biological activity, which was instead assessed through the SH-SY5Y-based assays.

Future studies should evaluate the effects of Calm under longer exposure periods and disease-related experimental conditions. In addition, primary and secondary neuronal cultures or various neuronal models should be physiologically evaluated. In summary, this study provides a preliminary in silico and in vitro framework suggesting that Calm constituents may interact with antidepressant-related molecular targets, particularly SLC6A4 and NTRK2. In addition, short-term Calm exposure altered neuroplasticity-associated gene expression markers in SH-SY5Y cells. These findings should be considered preliminary and hypothesis-generating, requiring protein-level and functional validation.

## 5. Conclusions

In conclusion, this study establishes a preliminary in silico and in vitro framework demonstrating that Calm constituents effectively interact with key antidepressant-related molecular targets, particularly SLC6A4 and NTRK2, while modulating neuroplasticity-associated gene expression markers in SH-SY5Y cells. Although short-term exposure altered transcriptional markers and enhanced metabolic activity at optimal doses, these preliminary data serve primarily as a hypothesis-generating foundation. Future independent investigations under extended exposure periods, comprehensive disease-related models, and protein-level functional assays are required to definitively validate the translation of these molecular interactions into therapeutic neuropharmacological outcomes.

## Figures and Tables

**Table 1:** Primer pairs used in the RT-PCR analysis. *GAPDH* was used as reference gene.

Gene	Primer Pairs
***BDNF***	F: 5′-TGGTTGCATGAAGGCTGCCC-3′ R: 5′-CATTCACGCTCTCCAGAGTCCC-3′
***NTRK2***	F: 5′-GAGCCTAACAGTGTAGATCC-3′ R: 5′-TTCTCAGTCCCACATAAGC-3′
***SYN1***	F: 5′-GAACAGGCCGAATTCTCTGA-3′ R: 5′-AACTGCGGTAGTCTCCGTTG-3′
***GAPDH***	F: 5′-CCACCCATGGCAAATTCC-3′ R: 5′-TGGGATTTCCATTGATGACAAG-3′

**Table 2:** Molecular docking interactions of reference antidepressants and Calm-derived compounds with selected neuropharmacological targets. Binding energies and predicted molecular interactions of sertraline, fluoxetine, and Calm constituents with SLC6A4, MAOA, AKT1 and NTRK2 protein structures are shown. Binding affinities are expressed as kcal/mol, with more negative values indicating stronger predicted ligand–protein interactions. Hydrogen bonds, hydrophobic interactions, salt bridges, and π-cation interactions were recorded based on the predicted ligand–protein binding poses. Numbers in parentheses indicate repeated interactions with the same amino acid residue.

	Ligand	Binding Energy (kcal/mol)	H-Bond	Hydrophobic Interactions	Salt Bridges	π-Cation Interactions
**NTRK2 (4AT3)**	**Sertralin**	−10.2	No	PHE565(2), VAL568(2), ALA586, MET636, LEU699(2)	No	PHE565
	**Fluoxetine**	−8.7	ASP710(2)	PHE565(2), VAL568(2), ALA586, LYS588, VAL617(2), LEU699	No	PHE633
	**Valerenic Acid**	−9.3	ASP710	PHE565(3), VAL568, VAL617, PHE633(4)	LYS588	No
	**Harmine**	−8.2	No	PHE565, LYS588(2), PHE633, LEU699	No	PHE565(2)
	**Apigenin**	−9.6	LYS588, ASP710, MET713	PHE565, VAL568(2), PHE633, LEU699(2), MET713	No	PHE565
	**5-Hidroksitriptofan**	−7.4	GLY709, MET713	PHE633, LEU699, ASP710	LYS588	PHE565
	**Rosmarinic Acid**	−9.4	LYS588, MET636(3), GLY639, MET713	LEU560, LYS588(2), PHE633(2), LEU699	No	No
	**Xanthohumol**	−8.7	LYS588, GLU604	LEU560, PHE565(2), VAL568, LYS588, PHE633, LEU699	No	LYS588
	**Safranal**	−6.4	LYS588	VAL568, LYS588, VAL617, PHE633(6), LEU699	No	No
	**Piridoksal5-fosfat**	−6.6	ASP710, MET713	PHE565, LEU699	LYS588	PHE565
**AKT1 (1H10)**	**Sertralin**	−5.3	GLU17	GLU85	No	LYS20
	**Fluoxetine**	−5.4	ALA50	TYR38, 39LYS(2), GLN47(2), LEU52	No	No
	**Valerenic acid**	−5.4	ASN53	GLU17, TYR18(2)	LYS14, ARG23, ARG25	No
	**Harmine**	−4.9	LYS14, ARG86	GLU17(2), TYR18	No	No
	**Apigenin**	−5.7	LYS20, THR65, GLU85	ARG15, ILE74, GLU85, THR87	No	No
	**5-Hidroksitriptofan**	−5.5	GLU17, THR65(2), THR87	ARG15, THR87	No	ARG15
	**Rosmarinic Acid**	−5.6	GLU17, TYR18, ILE19, ARG86	GLU17(2), TYR18	LYS14, ARG23(2)	No
	**Xanthohumol**	−5.8	LYS14, ARG23(2), ASN53, ARG86	GLU17, TYR18	No	No
	**Safranal**	−4.2	THR87	ARG15(2), LYS20	No	No
	**Piridoksal5-fosfat**	−5.5	GLU17, TYR18, ASN53,ARG86	GLU17(2)	LYS14, ARG23, ARG25	No
**MAOA (2Z5Y)**	**Sertralin**	−5.4	GLN215	TYR69(2), ILE180, PHE208, VAL210, GLN215(2), ILE335, LEU337(2), PHE352	No	PHE208
	**Fluoxetine**	−6.7	No	VAL210, GLN215, ILE335(2), PHE352, TYR407	No	No
	**Valerenic Acid**	−4.3	TYR407	PHE208, VAL210, GLN215, ILE335(2), LEU337(2), PHE352	No	No
	**Harmine**	−8.6	No	ILE180, PHE208, GLN215, ILE335, LEU337, PHE352, TYR407(2)	No	No
	**Apigenin**	−8.3	ASN181, TYR407	ILE180, GLN215, ILE335, LEU337(2), TYR407	No	PHE208
	**5-Hidroksittriptofan**	−7.8	ASN181, 208PHE	GLN215, ILE335, TYR407	No	No
	**Rosmarinic Acid**	−7.5	ALA111, ASN181, VAL210	PHE208, VAL210, ILE325, ILE335, LEU337, PHE352	No	TYR407
	**Xanthohumol**	−6.9	PHE208, GLY443	LEU97, ILE180, PHE208, VAL210, ILE325, ILE335, LEU337, TYR407, TYR444(2)	No	No
	**Safranal**	−5.6	GLN215	TYR69, ILE180, GLN215, ILE335, PHE352, TYR407, TYR444	No	No
	**Piridoksal5-fosfat**	−6.9	PHE208, GLN215	ILE180, GLN215, ILE335	No	No
**SLC6A4 (5I6X)**	**Sertralin**	−9.7	TYR95	TYR95, ILE172, TYR176, PHE341	No	TYR176
	**Fluoxetine**	−9.2	ASP98	ILE172(2), TYR176, PHE341, VAL501	No	No
	**Valerenic Acid**	−8.2	NO	ILE172(2), TYR176, PHE341(3)	No	No
	**Harmine**	−8.3	NO	TYR95, ILE172, TYR176(2)	No	TYR176
	**Apigenin**	−10.0	TYR95, ALA96, ASN177, SER438	ILE172(2), TYR176, PHE341	No	TYR176
	**5-Hidroksitriptofan**	−7.7	TYR95	TYR95, ILE172, ALA173, TYR176, PHE341	No	No
	**Rosmarinic Acid**	−9.0	ASP98, ASN177, SER439, THR497	TYR95, ILE172(2), TYR176, PHE335, PHE341	No	No
	Xanthohumol	−9.2	THR497(2)	ALA169, ILE172, PHE335(2), PHE341, VAL501	No	No
	Safranal	−5.7	NO	ILE172, TYR176, PHE341(2)	No	No
	Piridoksal5-fosfat	−6.7	SER(2)	TYR95	No	No

## Data Availability

The raw data supporting the conclusions of this article will be made available by the authors on request.
